# Disease trajectories in elders with suspected non-Alzheimer’s pathophysiology and its comparison with Alzheimer’s disease pathophysiology: a longitudinal study

**DOI:** 10.21203/rs.3.rs-2744271/v1

**Published:** 2023-03-29

**Authors:** Jie-Qiong Li, Jing-Hui Song, John Suckling, Yan-Jiang Wang, Chuan-Tao Zuo, Can Zhang, Jing- Gao, Yu-Qiang Song, An-Mu Xie, Lan Tan, Jin-Tai Yu

**Affiliations:** Hospital of Qingdao University; Hospital of Qingdao University; University of Cambridge; Daping Hospital, Third Military Medical University; Fudan University; Massachusetts General Hospital, Harvard Medical School; Hospital of Qingdao University; Hospital of Qingdao University; Hospital of Qingdao University; Qingdao University; Huashan Hospital, Fudan University

**Keywords:** suspected non-Alzheimer’s pathophysiology, biomarker, beta-amyloid, tau, Alzheimer

## Abstract

**Background:**

According to the new ‘AT(N)’ system, those with a normal amyloid biomarker but with abnormal tauopathy or biomarkers of neurodegeneration or neuronal injury, have been labeled suspected non-Alzheimer’s pathophysiology (SNAP). We aimed to estimate the long-term clinical and cognitive trajectories of SNAP individuals in non-demented elders and its comparison with individual in the Alzheimer’s disease (AD) pathophysiology using ‘AT(N)’ system.

**Methods:**

We included individuals with available baseline cerebrospinal fluid (CSF) Aβ (A), CSF phosphorylated tau examination (T) and 18F-uorodeoxyglucose PET or volumetric magnetic resonance imaging (N) from the Alzheimer’s Disease Neuroimaging Initiative database. Longitudinal change in clinical outcomes are assessed using linear mixed effects models. Conversion risk from cognitively normal (CN) to cognitively impairment, and conversion from mild cognitive impairment (MCI) to dementia are assessed using multivariate Cox proportional hazard models.

**Results:**

Totally, 366 SNAP individuals were included (114 A−T−N−, 154 A−T + N−, 54 A−T−N + and 44 A−T + N+) of whom 178 were CN and 188 were MCI. Compared with A−T−N−, CN elders with A−T + N−, A−T−N + and A−T + N + had a faster rate of ADNI-MEM score decline. Moreover, CN older individuals with A−T + N + also had a faster rate of decline in ADNI-MEM score than those with A−T + N− individuals. MCI patients with A−T + N + had a faster rate of ADNI-MEM and ADNI-EF decline and hippocampal volume loss compared with A−T−N− and A−T + N− profiles. CN older individuals with A−T + N + had an increased risk of conversion to cognitive impairment (CDR-GS ≥ 0.5) compared with A−T + N− and A−T−N−. In MCI patients, A−T + N + also had an increased risk of conversion to dementia compared with A−T + N− and A−T−N−. Compared with A−T + N−, CN elders and MCI patients with A + T + N− and A + T + N + had a faster rate of ADNI-MEM score, ADNI-EF score decline, and hippocampal volume loss. CN individuals with A + T + N + had a faster rate of ADNI-EF score decline compare with A−T + N + individuals. Moreover, MCI patients with A + T + N + also had a faster rate of decline in ADNI-MEM score, ADNI-EF score and hippocampal volume loss than those with A−T + N + individuals.

**Conclusions:**

The findings from clinical, imaging and biomarker studies on SNAP, and its comparison with AD pathophysiology offered an important foundation for future studies.

## Background

The term “suspected non-Alzheimer’s pathophysiology (SNAP)” were first proposed by Jack et al (1), soon after the publication of the 2011 National Institute on Aging–Alzheimer’s Association’s (NIA-AA) criteria, which indicated that individuals with imaging/biomarker evidence of Alzheimer’s disease (AD)-like neurodegeneration without β-amyloidosis. (2). By updating the 2011 guidelines which biomarkers were grouped into just two categories, in 2018, the NIA-AA published an updated research framework. It grouped biomarkers into three categories: 1) biomarkers of amyloid-β (Aβ) plaques (labeled “A”): cortical amyloid PET ligand binding or low CSF Aβ42; 2) biomarkers of paired helical filament tau (labeled “T”): elevated CSF phosphorylated tau (p-tau) and cortical tau PET ligand binding; 3) biomarkers of neurodegeneration or neuronal injury (labeled “N”): elevated CSF total tau (t-tau), 18F- fluorodeoxyglucose (FDG) PET, and brain atrophy on MRI (3). According to the new research framework, individuals are placed into the “Alzheimer’s continuum” if they have abnormal biomarkers of Aβ. Otherwise, those with a normal amyloid biomarker but with abnormal T or (N), or both, have been labeled SNAP. Therefore, three biomarker profiles (A−T−(N)+, A−T+(N)−, or A−T+(N)+) would be classified as SNAP.

We postulate that markers of non-AD pathologic processes corresponds to neurodegeneration due to a heterogeneous group of non-Alzheimer’s disease pathologies such as α-synucleinopathy, TDP 43, argyrophilic grains and hippocampal sclerosis. The A − T + N − profile corresponds to individuals have numerous NFTs which are similar to those in AD with the absence or scarcity of amyloid plaques throughout the brain (4). This presentation has been referred to as “atypical AD” (5, 6), and is considered as a disease entity different from “classical” AD (7). Until 2014, Crary et al. proposed a new term of “primary age-related tauopathy” (PART) to describe this pathological state as a way of facilitating communication among pathologists, clinicians, and researchers (8). The A − T + N + profile might correspond to a combination of primary age-related tauopathy and other non-Alzheimer’s disease pathologies.

Recently, we published a research revealed a pretty high proportion of SNAP in non-demented elderly individuals. However, the longitudinal outcomes of SNAP were absent (9). In previous researches, clinical and cognitive trajectories of SNAP were studied using A/N system (10–12) and urgently requires to be thoroughly analyzed based on the AT(N) system.

Herein, in this cohort study, we defined SNAP biologically according to the AT(N) system and studied the clinical outcomes longitudinally in a non-demented cohort from the Alzheimer’s Disease Neuroimaging Initiative (ADNI) database. Moreover, we studied the clinical outcomes in patients labeled SNAP on the basis of FDG-PET and MRI separately.

## Methods

### ADNI Study Design

We undertook cross-sectional and longitudinal analyses of participants enrolled in the ADNI database (adni.loni.usc.edu). The ADNI was launched in 2003 as a public-private partnership with the primary goal of testing whether serial MRI, PET, other biological markers, and clinical and neuropsychological assessments can be combined to measure the progression of mild cognitive impairment (MCI) and early AD. For up-to-date information on ADNI, see www.adni-info.org.

### Participants

We included all cognitively healthy controls and patients with MCI with the available ATN data, including the baseline CSF Aβ analysis (A), CSF p-tau examination (T) and FDG-PET or volumetric magnetic resonance imaging (MRI) (N) from the ADNI database. Detail information of the included participants were presented in the e-Methods in the Supplement. Amyloid abnormal (A+) and normal (A−) were determined by applying a cutoff value of 192pg/ml for CSF Aβ42 levels (13). Whether tau pathology was abnormal (T+) or normal (T−) used 23 pg/ml for CSF p-tau level (13). The cutoff point for FDG PET (N) (average of angular, temporal, and posterior cingulate) was 1.21 (14). Abnormal N was also defined as hippocampal volume adjusted for total intracranial volume (HVa) (e-Method) of less than 6723 mm^3^ (13,15).

### CSF Measurements

CSF Aβ42 and p-tau were measured at the ADNI Biomarker Core Laboratory (University of Pennsylvania) using the multiplex xMAP Luminex platform (Luminex Corp, Austin, TX) with Innogenetics (INNO-BIA AlzBio3; Ghent, Belgium; for research use-only reagents) immunoassay kit-based reagents. All CSF biomarker assays were performed in duplicate and averaged (13).

### Neuroimaging And Cognition

Amyloid PET imaging was measured with florbetapir. The global 18F- florbetapir standardized uptake value ratio (SUVR) was calculated by averaging the 18F-florbetapir retention ratio from four large cortical grey matter regions (frontal, anterior cingulate, precuneus and parietal cortex) using the cerebellum as a reference region.

FDG-PET data were acquired and reconstructed according to a standardized protocol (http://adni.loni.ucla.edu/). Spatial normalization of each individual’s PET volume to the standard template was conducted using SPM529. For FDG-PET, we averaged counts of angular, temporal, and posterior cingulate regions.

Structural MRI was performed using a Siemens Trio 3.0T scanner or Vision 1.5T scanner. Regional volume estimates were processed using Free-surfer software package version 4.3 and 5.1 image processing framework for the 1.5T and 3-T MRI images, respectively. Regions of interests (ROIs) included the hippocampus. Estimated intracranial volume (ICV) was used to adjust ROIs for head size variation based on a covariance approach as described.

Composite scores for executive functioning (ADNI-EF) and memory (ADNI-MEM) using data from the ADNI neuropsychological battery using item response theory (IRT) methods. The model for calculating ADNI-EF included Category Fluency-animals, Category Fluency-vegetables, Trails A and B, Digit span backwards, WAIS-R Digit Symbol Substitution, and 5 Clock Drawing items (circle, symbol, numbers, hands, time). The ADNI-MEM was composed of different word lists in the Rey Auditory Verbal Learning Test (RAVLT), the ADAS-Cog and Logical Memory I data missing by design.

### Statistical analysis

Differences across the three biomarker profiles, including A−T−N−, A−T + N− and A−T + N + were tested by the Kruskal-Wallis tests for continuous variables and chi-square tests for categorical data. To evaluate how clinical outcomes changed overtime, we used linear mixed-effects models. To access the risk of progression from cognitively normal (CN) to cognitively impairment indicated by the CDR–Global Score (CDR-GS) of 0.5 or greater, and from MCI to incident dementia, we constructed un-adjusted Kaplan-Meier plots. Additionally, we ran multivariate Cox proportional hazard models. Hazard ratios (HR) along with their 95% CIs were reported (e-Method). Differences of clinical outcomes at baseline and longitudinally among *MAPT* H1c genotypes were tested respectively by multi-factor analysis of variance and mixed-effect models, both of which were adjusted for age, gender and education (for cognition) or intracranial volume (for hippocampal volume). All statistical analyses were performed using the R statistical software (version 3.4.4).

## Results

### Baseline Characteristics

By using FDG to define N, 366 participants were classified as SNAP (114 A−T−N−, 154 A−T + N−, 54 A−T−N + and 44 A−T + N+) of whom 178 were CN and 188 were MCI. The participant characteristics were described in the Table 1. The mean (SD) age of the participants was 71.52 (7.03) years, 53.0% were men, and 98.6% had more than 12 years of education. Age, MMSE and Clinical Dementia Rating scale sum of boxes (CDRSB), ventricular and hippocampal volume and FDG-PET were significantly different among A−T−N−, A−T + N−, A−T−N + and A−T + N + profiles in the whole cohort (Table 1).

**Figure 1:**
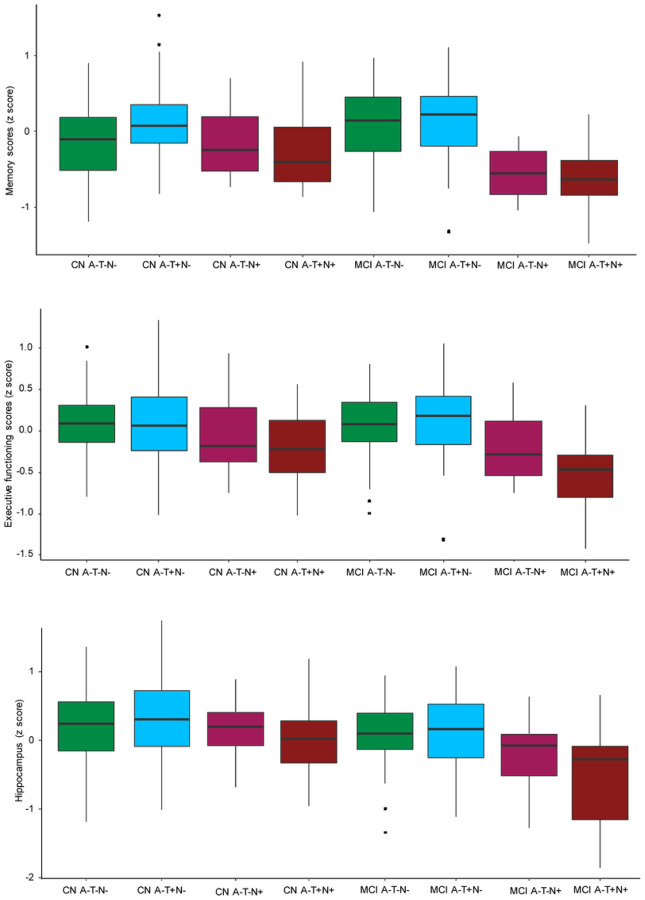
Hippocampal volume and cognitive measures across ATN profiles at baseline among non-demented older individuals. The box plot whiskers extend to the lowest and highest data points within 1·5 times the inter-quartile range from the lower and upper quartiles. The dots represent individual points that fall outside this range. MCI=mild cognitive impairment. CN=cognitively normal. A−=amyloid normal. T−=tau normal. T+=tau abnormal. N−=neurodegeneration or neuronal injury normal. N+=neurodegeneration or neuronal injury abnormal.

In the CN group, hippocampal volume, ADNI-MEM and ADNI-EF did not differ significantly among the four profiles after adjusted for age, gender, ICV (for hippocampal volume) and years of education (for cognitive measures). However, in MCI group, hippocampal volume differed (P = 0.004) with A−T + N + smaller than A−T−N− (P = 0.003) and A−T + N− (P = 0.003), A−T−N + smaller than A−T−N− (P = 0.02). In addition, ADNI-MEM (P < 0.001) and ADNI-EF (P = 0.007) both differed among the four profiles with A−T + N + having lower scores than A−T−N− (P = 0.001 in ADNI-MEM and P = 0.007 in ADNI-EF) and A−T + N− (P = 0.007 in ADNI-MEM and P = 0.003 in ADNI-EF), A−T−N + having lower scores than A−T−N− (P = 0.01 in ADNI-MEM and P = 0.02 in ADNI-EF) (Fig. 1). Baseline characteristics of the included samples using HVa defining N was described in supplementary table 1 and supplementary Fig. 1–3.

### Longitudinal Change

By using FDG to define N, only hippocampal atrophy was observed in all the four profiles (A−T−N−: −1.09% [95% CI −1.4% to −0.78%], P < 0.001; A−T + N−: −1.13% [95% CI −1.37% to −0.89%], P < 0.001; A−T−N+: −1.39% [95% CI −2.02% to −0.76%], P < 0.001; A−T + N+: −1.65% [95% CI −2.38% to −0.93%], P < 0.001) in CN older individuals. Compared with A−T−N−, CN older individuals with A−T + N− (−0.02% (95% CI −0.04% to −0.001%), P = 0.04), A−T−N+ (−0.02% (95% CI −0.04% to −0.009%), P = 0.04) and A−T + N+ (−0.03% (95% CI −0.05% to −0.005%), P = 0.02) had a faster rates of ADNI-MEM score decline. Moreover, CN older individuals with A−T + N + also had a faster rate of decline in ADNI-MEM score than those with A−T + N− individuals (−0.06% [95% CI −0.11% to −0.01%], P = 0.009). However, the rate of ADNI-EF decline and hippocampal volume loss did not differ among the three profiles. Compared with A−T + N−, CN older individuals with A + T + N− (−0.05% (95% CI −0.08% to −0.02%), P = 0.004) and A + T + N+ (−0.08% (95% CI −0.12% to −0.03%), P = 0.001) had a faster rates of ADNI-MEM score, ADNI-EF score decline (A + T + N− ((−0.04% (95% CI −0.08% to −0.009%), P = 0.04), A + T + N+ (−0.14% (95% CI −0.2% to −0.08%), P < 0.001) and hippocampal volume loss (A + T + N− ((−0.6% (95% CI −1.19% to −0.007%), P = 0.048), A + T + N+ (−1.52% (95% CI −2.21% to −0.81%), P < 0.001). Moreover, CN older individuals with A + T + N + also had a faster rate of decline in ADNI-EF score than those with A−T + N + individuals (−0.1% [95% CI −0.19% to −0.008%], P = 0.03). (Supplementary table 4–5)

**Figure 2:**
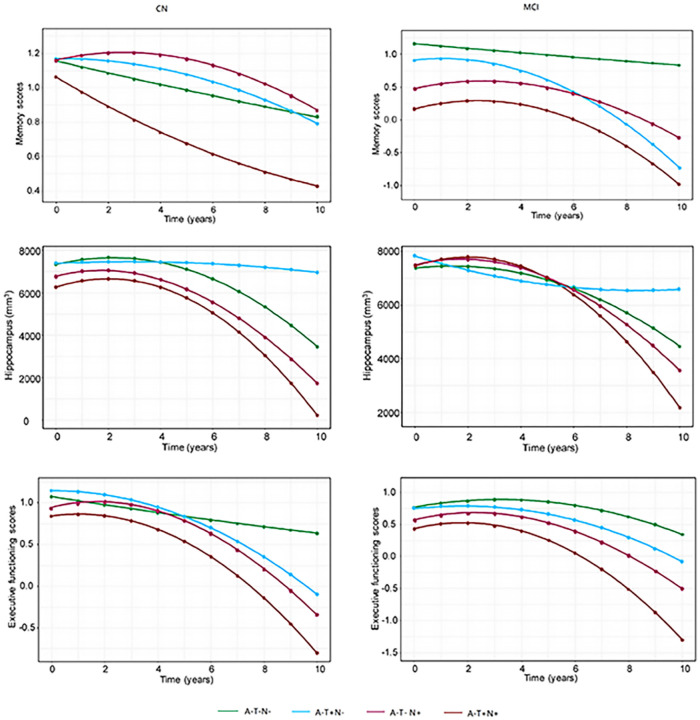
Models of longitudinal change in clinical outcomes based on linear mixed effects regression. Analyses of cognitive decline were adjusted for age, gender and education years. Analyses of brain atrophy were adjusted for age, gender, total intracranial volume and field strength (1·5T vs 3T). The P value indicated the differences among three profiles. FDG=18F-uorodeoxyglucose. MCI=mild cognitive impairment. CN=cognitively normal. A-=amyloid normal. T−=tau normal. T+=tau abnormal. N−=neurodegeneration or neuronal injury normal. N+=neurodegeneration or neuronal injury abnormal.

In MCI patients, hippocampal atrophy was observed in all the four profiles (A−T−N−: −1.07% [95% CI −1.56% to −0.57%], P < 0.001; A−T + N−: −1.09% (95% CI −1.52% to −0.66%), P < 0.001; A−T−N+: −1.89% (95% CI −2.17% to −1.61%), P < 0.001; A−T + N+: −2.08% [95% CI −2.89% to −1.25%), P < 0.001). Beyond that, ADNI-MEM (−0.06% [95% CI −0.11% to −0.02%], P = 0.005) and ADNI-EF decline (−0.06% [95% CI −0.11% to −0.003%], P = 0.04) was only observed in A−T + N+. MCI patients with A−T + N + had a faster rate of ADNI-MEM (A−T−N−: −0.06% [95% CI −0.11% to −0.01%], P = 0.01; A−T + N−: −0.08% [95% CI −0.13% to −0.03%], P = 0.001) and ADNI-EF decline (A−T−N−: −0.08% [95% CI −0.14% to −0.02%], P = 0.01; A−T + N−: −0.06% [95% CI −0.12% to −0.006%], P = 0.03) and hippocampal volume loss (A−T−N−: −1.01% [95% CI −1.96% to −0.04%], P = 0.04; A−T + N−: −0.99% [95% CI −1.92% to −0.05%], P = 0.04) compared with A−T−N− and A−T + N− profiles. A−T−N + showed faster ADNI-MEN, ADNI-EF decline and hippocampal volume loss than A−T−N−(Fig. 2 and supplementary table 2–3). Compared with A−T + N−, MCI patients with A + T + N− (−0.11% (95% CI −0.15% to −0.06%), P < 0.001) and A + T + N+ (−0.25% (95% CI −0.29% to −0.21%), P < 0.001) had a faster rates of ADNI-MEM score, ADNI-EF score decline (A + T + N− ((−0.1% (95% CI −0.15% to −0.04%), P < 0.001), A + T + N+ (−0.14% (95% CI −0.2% to −0.08%), P < 0.001) and hippocampal volume loss (A + T + N− ((−1.64% (95% CI −2.4% to −0.88%), P < 0.001), A + T + N+ (−2.53% (95% CI −3.26% to −1.79%), P < 0.001). Moreover, MCI patients with A + T + N + also had a faster rate of decline in ADNI-MEM score (−0.16% [95% CI −0.22% to −0.09%], P < 0.001), ADNI-EF score (−0.2% [95% CI −0.29% to −0.12%], P < 0.001) and hippocampal volume loss (−1.52% [95% CI −2.74% to −0.33%], P = 0.001) than those with A−T + N + individuals (Supplementary table 4–5). In addition, we examined the differences in the change rates of clinical outcomes in females versus males but did not detect any differences in either MCI or CN individuals (Supplementary table 6). Longitudinal Change using HVa to define N was shown in supplementary table 2–3 and supplementary Fig. 4–9.

### Risk Of Disease Progression

By using FDG to define N, CN older individuals with A−T + N + had an increased risk of conversion to cognitive impairment (CDR-GS ≥ 0.5) compared with A−T + N− (HR: 3.06, 95% CI 1.14 to 8.2, P = 0.026) and A−T−N− (HR: 3.19, 95% CI 1.06 to 9.6, P = 0.039). A−T−N + had an increased risk of conversion compared with A−T−N− (HR: 3.01, 95% CI 1.02 to 9.1, P = 0.041). In MCI patients, A−T + N + also had an increased risk of conversion to dementia compared with A−T + N− (HR: 4.54, 95% CI 1.12 to 18.3, P = 0.034) and A−T−N− (HR: 4.74, 95% CI 1.12 to 20.1, P = 0.035). A−T−N + had an increased risk of conversion compared with A−T−N− (HR: 4.11, 95% CI 1.11 to 17.9, P = 0.034). However, we did not detect any differences in conversion risk between A−T + N− and A−T−N−, in either CN or MCI groups (Table 2). Covariates of both models met proportional hazards assumptions using Schoenfeld residuals technique (Global Schoenfeld Test p = 0.45 and 0.63, respectively). Progression risk using HVa defining N was shown in supplementary table 7.

Compared with A−T + N−, CN older individuals with A + T + N−(HR: 2.27, 95% CI 1.2 to 4.3, P = 0.011) and A + T + N+ (HR: 4.21, 95% CI 2.08 to 8.5, P < 0.001) and had an increased risk of conversion to cognitive impairment (CDR-GS ≥ 0.5). Moreover, MCI patients with A + T + N−(HR: 18.9, 95% CI 2.57 to 138.8, P = 0.004) and A + T + N+ (HR: 69.7, 95% CI 9.67 to 501.6, P < 0.001) also had an increased risk of conversion to dementia compared with A−T + N−. MCI patients with A + T + N + also had an increased risk of conversion to dementia compared with A−T + N+(HR: 3.5, 95% CI 1.43 to 8.63, P = 0.0006) (supplementary table 8–9).

## Discussion

We performed analyses to examine baseline and longitudinal cognitive decline (memory and executive function) and hippocampal atrophy in SNAP individuals according to the ATN system. We also compare the cognitive trajectories in SNAP with AD pathophysiology. Our main findings were that using FDG to define N, hippocampal volume and cognition differed among A−T−N−, A−T + N−, A−T−N + and A−T + N + in MCI patients at baseline with the lowest volume and the worst performance displayed in A−T + N+. Furthermore, hippocampal atrophy was observed in all the four profiles either in CN or MCI groups, although memory impairment was only observed in A−T−N + and A−T + N+. Moreover, A−T + N + showed a faster rate of memory loss than A−T−N− and A−T + N−, and A−T−N + was faster than A−T−N−. In addition, executive function impairment was only observed in A−T + N + in MCI patients. A−T−N + and A−T + N + had increased risks of conversion from CN to cognitively impairment, and conversion from MCI to dementia, compared with A−T−N− and A−T + N−. Compared with A−T + N−, A + T + N− and A + T + N + had a faster rate of cognitive decline, and hippocampal volume loss. A + T + N + had a faster rate of executive function impairment compare with A−T + N + individuals.

To date, several published studies have described the change in clinical outcomes of SNAP. Based on the NIA-AA criteria for preclinical AD (2), Aβ−/NFT + could be categorized as suspected non-AD pathophysiology (SNAP) as high CSF tau (total or phosphorylated) is recognized as a biomarker of neurodegeneration. Among CN older individuals, the rates of cognitive decline and loss of hippocampal volume in individuals with SNAP are indistinguishable from those in the A−N− group (11, 12, 16, 17). However, other studies indicate that the SNAP group had a practice effect on cognitive performance over time compared with the A−N− group (10, 15). In contrast to the ATN system, the 2011 criteria placed both CSF p-tau and t-tau into the “N” group. Researchers increasingly this classification recognized method was inappropriate. CSF p-tau specifically reflects the phosphorylation state of tau, different from t-tau which reflects nonspecific neurodegeneration or neuronal injury (18). Therefore, the “ATN” scheme separates biomarkers of pathologic tau (CSF p-tau) from measures of neurodegeneration or neuronal injury. A−N+ (SNAP) according to 2011 NIA-AA criteria could encompass A−T + N−, A−T + N + and A−T−N + according to the ATN system. Consistent with previous studies, we did not detect any difference in the rate of cognitive decline and loss of hippocampal volume between A−T + N− and A−T−N−. However, A−T−N + and A−T + N + showed a faster rate of cognitive decline and hippocampal atrophy than A−T−N−. One explanation is that “N” in SNAP according to 2011 NIA-AA criteria is defined as CSF p-tau, which could be A−T + N− in our research.

PART is defined by AD-type neurofibrillary changes without, or with few Aβ plaques detected at autopsy (8). NFTs in PART, detected by immunohistochemical and biochemical studies, contain both 3-repeat (3R) and 4-repeat (4R) tau isoforms, identical to those in classical AD (7). CSF Aβ and p-tau are considered as biomarkers of two pathological states that are associated with amyloid protein and paired helical filament tau formation respectively in brains. Therefore, it is reasonable to conclude that the A − T + N ± profiles correspond to PART, and that A−T + N + represents a relatively severe stage. However, common non-AD, non-PART tauopathy (Aging-related tau astrogliopathy) as well as non-tauopathic causes of neurodegeneration, particularly limbic-predominant age-related TDP-43 proteinopathy are definitely confounders, and their various risks seem to change along the aging spectrum (19, 20). The proportion of observed tauopathy and neurodegeneration that can be attributed to PART versus other possible comorbid conditions is unknown. The finding of “T” and “N” biomarkers (FDG and hippocampal volume) were not specific to PART. A helpful solution to this non-specificity is to reveal the contribution of tau to neurodegeneration in participants labeled PART on the basis of MRI and FDG-PET in longitudinal studies.

Hippocampal atrophy was observed in all the four profiles. However, cognitive decline was only observed in A−T−N + and A−T + N+. This could be partly explained by brain atrophy occurring earlier than cognitive decline (21, 22). We also observed that memory impairment occurred earlier when HVa was used to define N compared with FDG-PET. This may suggest that hippocampal atrophy has a greater influence on memory compared with low glucose metabolism, which is consistent with previous studies (23, 24). Moreover, executive function impairment was only observed in A−T + N + when FDG-PET was used to define N. This suggested that the low glucose metabolism may influence executive function greater than hippocampal atrophy does. Most studies report that individuals with PART usually display normal cognition or a lesser degree of cognitive impairment. We detected cognitive decline only in the severe stages of PART. However, in the early stages of PART, although cognitive decline does not occur, the hippocampus begins to atrophy. Cognitive impairment is likely to follow.

Numerous studies have confirmed the association between NFTs and cognitive status. Prior studies also suggested that increased NFT burden is correlated with cognitive decline and hippocampal atrophy in PART (8, 25–27). However, in our study, we did not detect significant differences in the rates of cognitive decline or hippocampal atrophy between A−T−N ± and A−T + N ± profiles. An explanation for this inconsistency may be that previous work did not take neurodegeneration into account whether severe NFT burden is accompanied by neurodegeneration is unclear. Additionally, NFT was detected on strictly neuropathological criteria in previous studies, but we used CSF p-tau to reflect NFT burden in the brain. The correlation between CSF p-tau and brain NFT burden is imperfect, especially in later stages of the disease as the CSF p-tau plateaus whereas NFT burden continues to increase (28).

Based on the NIA-AA criteria for preclinical AD (2), researchers studied the difference in the progression risk between cognitively normal individuals (no amyloid, no neuronal injury, no subtle cognitive decline) and SNAP individuals (abnormal CSF t-tau or p-tau in the presence of normal Aβ, regardless of episodic memory ability). As a result, they did not find any difference in the risk of progression from CN to CDR at least 0.5 between SNAP and normal individuals (17, 29). In MCI patients, prior studies indicated that SNAP patients had significantly higher risk of progressive cognitive deterioration compared with the normal (A−N−) group (30, 31). Consistently, we detected that A−T−N + and A−T + N + had significant increased risk from MCI to dementia than A−T−N−; however, we did not detect any difference between A−T−N− and A−T + N− in progression risk from CN to CDR at least 0.5 and from MCI to dementia.

Previous studies showed inconsistent results in the comparison of cognitive trajectories between SNAP and AD pathophysiology. (10–12, 30, 32). Different from their A/N framework, we used the A/T/N framework. We found that, in the same status of baseline tau pathology and neurodegeneration (T and N), the presence of baseline amyloid could accelerate cognitive decline and clinical progression. Although results were distinct, a majority of studies indicated that high Aβ deposition in elders was associated with decline in cognition in the decade (33–35), which consistent with our findings.

## Conclusion

We described the entire clinicopathological spectrum of SNAP individuals. We also estimated its comparison with Alzheimer’s continuum individuals with longitudinal clinical data. In doing so, we provide the clinical, imaging, and biomarker communities involved in the diagnosis and treatment of SNAP with an important foundation for future studies.

## Figures and Tables

**Table 1.** Baseline Demographic Characteristics of Entire Cohort Using FDG to define N*

Biomarker profiles (n)	A−T−N− (114)	A−T+N− (154)	A−T−N+(54)	A−T+N+(44)	P value†
**AGE(mean(sd))**	70.64 (6.45)	70.50 (7.14)	74.05 (6.96)	74.24 (6.86)	<0.001
**Male sex, No. (%)**	56 (49.1)	76 (49.4)	35 (64.8)	27 (61.4)	0.123
**Educational level, y**	16.38 (2.76)	16.68 (2.61)	15.96 (2.66)	16.30 (2.68)	0.378
**MMSE score**	28.75 (1.38)	28.86 (1.24)	28.57 (1.64)	28.16 (1.79)	0.034
**CDRSB score**	0.72 (0.83)	0.47 (0.68)	0.95 (0.88)	0.80 (0.94)	0.001
**Ventricles volume, mm3**	33812.67 (18984.09)	26362.66 (12742.51)	41036.65 (20928.77)	42703.25 (26011.91)	<0.001
**Hippocampus volume, mm3**	7502.04 (865.46)	7557.94 (960.54)	7203.07 (1082.77)	7126.55 (1476.67)	0.04
**Amyloid PET**	1.02 (0.07)	1.03 (0.07)	1.01 (0.07)	1.02 (0.06)	0.484
**FDG PET**	1.37 (0.09)	1.36 (0.08)	1.18 (0.05)	1.19 (0.06)	<0.001

Abbreviations: A−, amyloid normal. T−, tau normal. T+, tau abnormal. N−, neurodegeneration or neuronal injury normal. N+, neurodegeneration or neuronal injury abnormal. CDRSB, Clinical Dementia Rating scale sum of boxes. CSF, cerebrospinal fluid; FDG=18F-fluorodeoxyglucose. HVa= hippocampal volume adjusted for total intracranial volume. MCI, mild cognitive impairment; MMSE, Mini-Mental State Examination; ND, neuronal degeneration; PET, positron emission tomography; pTau, phosphorylated tau.

* Unless otherwise indicated, data are expressed as mean (SD)

† p value is for difference among three profiles based on one-way t test for continuous variables, χ^2^ testing for categorical variables and Kruskal-Wallis χ^2^ test for the non-normal length of follow-up variable.

**Table 2.** Progression risk from CN to cognitively impairment and from MCI to dementia

	Progression from CN to cognitively impairment				Progression from MCI to dementia			
	Hazard ratio (95% CI) *	P-value	Hazard ratio (95% CI) *	P-value	Hazard ratio (95% CI) *	P-value	Hazard ratio (95% CI) *	P-value
A−T−N−	Reference		/	/	Reference		/	/
A−T+N−	1.21(0.50–3.0)	0.673	Reference		1.23(0.27–5.5)	0.792	Reference	
A−T−N+	3.01(1.02–9.1)	0.041	2.48(0.91–8.1)	0.08	4.11(1.11–17.9)	0.034	4.22(0.98–16.8)	0.057
A−T+N+	3.19(1.06–9.6)	0.039	3.06(1.14–8.2)	0.026	4.74(1.12–20.1)	0.035	4.54(1.12–18.3)	0.034

Abbreviations: CN, cognitively normal; MCI, mild cognitive impairment; A−, amyloid normal; T−, tau normal; T+, tau abnormal; N−, neurodegeneration or neuronal injury normal; N+, neurodegeneration or neuronal injury abnormal.

* Hazard ratios (95% CI) calculated using Cox regression analyses and corrected for baseline age, gender and years of education.

## Abbreviations

AD: Alzheimer’s disease
ADNI: Alzheimer’s Disease Neuroimaging Initiative
CSF: cerebrospinal fluid
CN: cognitively normal
CN: cognitively normal
CDR-GS: CDR–Global Score
FDG: fluorodeoxyglucose
HR: Hazard ratios
ICV: intracranial volume
MCI: mild cognitive impairment
MRI: magnetic resonance imaging
NIA-AA: National Institute on Aging–Alzheimer’s Association’s
PART: primary age-related tauopathy”
ROIs: Regions of interests
RAVLT: Rey Auditory Verbal Learning Test
SNAP: suspected non-Alzheimer’s pathophysiology
SUVR: standardized uptake value ratio
